# Elevated preoperative Galectin-3 is associated with acute kidney injury after cardiac surgery

**DOI:** 10.1186/s12882-018-1093-0

**Published:** 2018-10-20

**Authors:** Moritz Wyler von Ballmoos, Donald S. Likosky, Michael Rezaee, Kevin Lobdell, Shama Alam, Devin Parker, Sherry Owens, Heather Thiessen-Philbrook, Todd MacKenzie, Jeremiah R. Brown

**Affiliations:** 1 Division of Cardiovascular and Thoracic Surgery, Duke University Medical Center, Durham, NC USA; 2 Institute for Healthcare Policy and Innovation, University of Michigan, Ann Arbor, MI USA; 3 Section of Health Services Research and Quality, Department of Cardiac Surgery, University of Michigan, Ann Arbor, MI USA; 4 Section of Urology, Department of Surgery, Dartmouth-Hitchcock Medical Center, Lebanon, NH USA; 5 Carolinas HealthCare System, Charlotte, NC USA; 6 The Dartmouth Institute for Health Policy and Clinical Practice, Geisel School of Medicine, Lebanon, NH USA; 7 Division of Nephrology, Department of Medicine, Johns Hopkins University, Baltimore, MD USA; 8 Department of Biomedical Data Science, HB 7505 Dartmouth-Hitchcock Medical Center, Lebanon, NH NH 03756 USA; 9 Department of Epidemiology, Geisel School of Medicine, Lebanon, NH USA

**Keywords:** Acute kidney injury (AKI), Galectin-3 (Gal-3), Cardiac surgery, Prediction, Biomarkers

## Abstract

**Background:**

Previous research suggests that novel biomarkers may be used to identify patients at increased risk of acute kidney injury following cardiac surgery. The purpose of this study was to evaluate the relationship between preoperative levels of circulating Galectin-3 (Gal-3) and acute kidney injury after cardiac surgery.

**Methods:**

Preoperative serum Gal-3 was measured in 1498 patients who underwent coronary artery bypass graft (CABG) surgery and/or valve surgery as part of the Northern New England Biomarker Study between 2004 and 2007. Preoperative Gal-3 levels were measured using multiplex assays and grouped into terciles. Univariate and multinomial logistic regression was used to assess the predictive ability of Gal-3 terciles and AKI occurrence and severity.

**Results:**

Before adjustment, patients in the highest tercile of Gal-3 had a 2.86-greater odds of developing postoperative KDIGO Stage 2 or 3 (*p* < 0.001) and 1.70-greater odds of developing KDIGO Stage 1 (*p =* < 0.001), compared to the first tercile. After adjustment, patients in the highest tercile had 2.95-greater odds of developing KDIGO Stage 2 or 3 (*p <* 0.001) and 1.71-increased odds of developing KDIGO Stage 1 (*p* = 0.001), compared to the first tercile. Compared to the base model, the addition of Gal-3 terciles improved discriminatory power compared to without Gal-3 terciles (test of equality = 0.042).

**Conclusion:**

Elevated preoperative Gal-3 levels significantly improves predictive ability over existing clinical models for postoperative AKI and may be used to augment risk information for patients at the highest risk of developing AKI and AKI severity after cardiac surgery.

## Background

Acute kidney injury (AKI) is a well recognized complication following cardiac surgery, and significantly affects morbidity and mortality [1, 2]. Up to 40% of patients develop AKI after cardiac surgery and places patients at 5-fold higher risk of death during hospitalization [3]. AKI has also been associated with hospital readmissions following cardiac surgery and hospitalization for heart failure or acute myocardial infarction (MI) [4–8]. Conventional metrics used to define and monitor the progression of AKI, such as serum creatinine and blood urea nitrogen levels, are insensitive, nonspecific and change notably only after significant kidney injury [8]. Identifying patients at increased risk of AKI prior to surgery is critical to take preventative measures and counsel patients on potential outcomes after cardiac surgery. A timely diagnosis would allow for earlier clinical intervention, greater care management prior to surgery, improved patient engagement and could improve patient outcomes.

New biomarkers allow a diagnosis to be made earlier than conventional measures, and allows kidney injury to be diagnosed even in the absence of concurrent or subsequent dysfunction. Biomarkers have been utilized to investigate AKI and augment the prediction of AKI risk and other complications following cardiac surgery [9–12]. A specific and sensitive marker of AKI risk could improve risk stratification, potentially identify patients that will benefit from greater care management prior to surgery and alert clinicians to individuals that will need earlier interventions to prevent AKI. Current risk prediction models for AKI following cardiac surgery have been developed on patient and disease characteristics alone. The addition of a specific protein biomarker may improve predictive ability over existing clinical models and may augment risk information for patients at higher risk of AKI after surgery.

Galectin-3 (Gal-3) is a beta-galactoside-binding lectin that has emerged as a key regulator of inflammation and tissue fibrosis [13]. Experimental studies in models of cancer, congestive heart failure and inflammatory disease have demonstrated that Gal-3 expression is elevated in these pathologic conditions [9]. In animal models, Gal-3 is acutely up-regulated in the kidneys in response to ischemic and toxic injury and is associated with renal fibrosis [14–17]. In humans, elevated levels of circulating Gal-3 have been found to be associated with increased risk of incident chronic kidney disease (CKD) and loss of kidney function over time [11, 18]. Gal-3 therefore can be considered a marker of both acute and chronic inflammatory processes in the kidneys, even in the absence of traditional clinical markers of renal injury.

There is an urgent need to analyze the predictive utility of Gal-3 to identify patients at greater risk of developing postoperative AKI. To date, Gal-3 has not been investigated as a potential biomarker for AKI in humans. The purpose of this study was to evaluate the assocation between preoperative Gal-3 and postoperative AKI in a large cohort of cardiac surgical patients.

## Methods

### NNE biomarker study

This study expands on the experience of Northern New England Cardiovascular Disease Study Group (NNECDSG), a regional collaborative consortium founded in 1987. All eight hospitals in this consortium submit data on cases with validation of procedure numbers and mortality conducted every 2 years. The NNECDSG registry contains data on patient characteristics, procedural indications, clinical variables and in-hospital outcomes. Data are periodically validated to ensure that all procedures and endpoints included in the registry have been accurately assessed. The NNECDSG has extensive experience in risk prediction in CABG surgery [4, 5]. The Northern New England (NNE) Biomarker Study is an initiative designed to assess the role of biomarkers in cardiac surgery.

Patient, procedural and outcome data were collected from patients undergoing coronary CABG surgery. Those undergoing CABG incidental to heart valve repair or replacement, resection of a ventricular aneurysm, or other surgical procedure were not included. Only patients that had biomarker levels collected were retained in the final analyses (*n* = 1498). For the present study, the sample included patients undergoing emergent, urgent and non-urgent CABG surgeries. Investigators and patients were blinded to the collected biomarker levels. The Committee for the Protection of Human Subjects at Dartmouth College (Institutional Review Board) approved this study for both the prospective cohort with patient consent and the linkage of readmission and mortality events.

### Galectin-3

Preoperative levels of Gal-3 was the main exposure of interest for this study. Blood samples were preoperatively collected prior to incision at each participating site in a 10-mL serum tube. Preoperative biomarker levels were measured using custom made multiplex ELISA assays (Meso Scale Discovery, Rockville, MD). Blood was allowed to clot at room temperature for 20 min to separate out the red blood cells, the tubes were centrifuged at 3500 rpm for 20 min, and the sera stored at the respective medical centers below − 80 degree Celcius until transportation on dry ice to the Laboratory for Clinical and Biomedical Research in Colchester, Vermont where they were stored at − 80 degree Celcius until measurement. Frozen serum was analyzed at a central laboratory, at the same time for biomarker measurement. Biomarkers were linked to the NNECDSG cohort to conduct the preoperative risk prediction modeling. Biomarkers were evaluated as continuous variables, natural log-transformed, and as terciles.

### Acute kidney injury

The primary outcome of this study was the development of AKI after cardiac surgery. The last serum creatinine (SCr) prior to cardiac surgery and highest postoperative SCr prior to discharge were used to classify the stage of AKI. AKI stages were defined by the Kidney Disease: Improving Global Outcomes (KDIGO) definition as follows [19]: Stage 1: increase in SCr by > 0.3 mg/dL within 48 h or > 1.5 times baseline within 7 days; Stage 2: increase in SCr to 2 to 2.9-fold baseline; and Stage 3: increase in SCr to 3.0 times baseline or increase in SCr to > 4.0 mg/dL or initiation of renal replacement therapy. Due to the small proportion of patients in KDIGO stage 2 and 3, we bundled stage 2 or 3 patients’ outcomes in this report.

### Statistical analysis

We evaluated the Gal-3 measurements to determine the association with the primary outcome (AKI) using univariate and multinomial logistic regression. Postoperative outcomes were compared using chi-squared tests, and continuous data were compared using the ANOVA test with the Bonferroni correction. Adjustment was carried out using variables from the Society of Thoracic Surgeons (STS) readmission model (Appendix) [20].To evaluate the association of the biomarker with AKI outcomes, we divided the cohort into terciles on the basis of Gal-3 values, where the lowest tercile is the reference group. We included indicator variables for the middle and highest terciles. We applied the mean imputation replacement method to account for variables with missing values. All biomarker values below the assay’s lower quantitative limit were assigned the lower limit of detection. The performance of the risk model was assessed by measuring the total area under the receiver operating characteristic curve (AUC or c-statistic). Standard errors and 95% confidence intervals were estimated for the c-statistic using a bootstrap method. All analyses were conducted using Stata 13.1 College Station, TX).

### Secondary Analyes

We compared the incremental value of Gal-3 to preoperative eGFR, a traditional risk marker of AKI. We adjusted the model using the STS readmission model and used the test of equality of ROC areas to assess differences in model performance. We calculated the Net Reclassification Improvement (NRI) and Integrated Discrimination Improvement (IDI) indices for risk models including Gal-3 and eGFR values.

Finally, we also created an alternative final model including both preoperative N-terminal pro b-type natriuretic peptide (NT-proBNP) values and preoperative Gal-3, in addition to the base STS readmission prediction model, to assess the predictive power using a combination of biomarkers. We compared model performance using the test of equality of ROC areas.

## Results

**Fig. 1:**
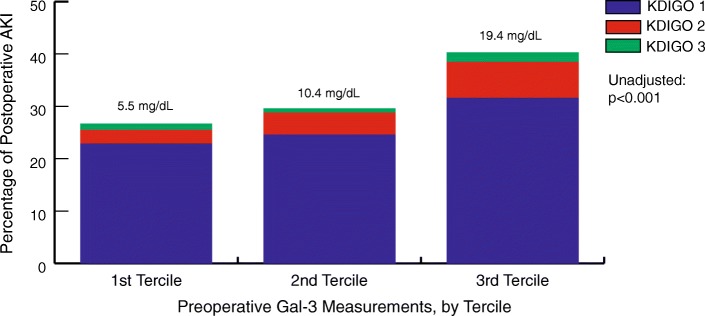
Association of preoperative Galectin-3 and postoperative AKI severity after coronary artery bypass graft surgery, by Galectin-3 tercile. The mean Galectin-3 measurement for each tercile increases stepwise with postoperative AKI severity. There is a significant relationship between elevated preoperative Galectin-3 and AKI severity by KDIGO

Overall, 1489 patients were included in the study. 481 (32.1%) developed AKI within KDIGO Stage 1 (26.3%) and 87 (5.8%) experienced KDIGO Stage 2 or 3 (Fig. 1). Patient and procedural characteristics are summarized in Table 1. Patient and procedural characteristics and the association with Gal-3 tercile measurements are summarized in Table 2. Gal-3 sample measurements ranged from 1.38 to 102.35 ng/mL with a median (Q1, Q3) value of 10.30 ng/mL (6.96 to 14.67 ng/mL).

**Table 1:** Patient and procedural characteristics and postoperative AKI occurrence

	No AKI (*N* = 1017)	AKI (*N* = 481)	*p* value
Age*	64.02 ± 9.99	68.08 ± 9.90	0.000
Female	23.8%	23.0%	0.740
BMI*	29.22 ± 5.17	30.35 ± 6.07	0.000
BSA*	2.03 ± 0.24	2.04 ± 0.26	0.200
Smoker	26.1%	16.6%	0.000
Atrial fibrillation	5.2%	9.3%	0.003
CHF	8.4%	13.5%	0.002
Pre-operative creatinine*	1.08 ± 1.02	1.15 ± 0.43	0.108
Diabetes	32.8%	43.3%	0.000
EF < 40	8.9%	14.0%	0.003
Hypertension	80.2%	81.7%	0.503
Pre-operative IABP	3.2%	5.7%	0.019
Prior MI			0.004
None	59.7%	49.8%	
< 24 h preop	1.5%	1.9%	
> 24 h & < 7 days	17.7%	21.9%	
> 7 & < 365 days	8.4%	12.5%	
> 365 days	12.7%	13.9%	
VAD	24.5%	32.5%	0.001
Unstable angina	55.3%	56.0%	0.780
COPD	12.4%	14.3%	0.321
LM stenosis	33.3%	35.0%	0.520
Prior CABG	1.8%	3.3%	0.064
Prior PCI	20.3%	18.4%	0.396
Priority			0.297
Emergency or emergent salvage	1.5%	2.7%	
Urgent	67.6%	67.9%	
Non-urgent	30.9%	29.5%	
Received transfused blood	29.1%	54.9%	0.000
pRBCs transfused pre-operatively			0.385
0	98.5%	97.3%	
1	0.4%	1.0%	
2	0.7%	1.3%	
3 or more	0.4%	0.4%	
pRBCs transfused post-operatively			0.000
0	77.8%	57.9%	
1	5.9%	9.1%	
2	10.7%	16.8%	
3 or more	5.7%	16.2%	
Pump time (mean, SD)	100.88 ± 32.01	112.44 ± 37.43	0.000

*AKI* acute kidney injury, *BMI* body mass index (kg/m^2^), *BSA* body surface area (m^2^), *CABG* coronary artery bypass graft, *CHF* congestive heart failure, *COPD* chronic obstructive pulmonary disease, *IABP* intra-operative balloon pump, *MI* myocardial infarction, *PCI* percutaneous coronary intervention, *RBC* red blood cell, *SD* standard deviation

*signifies continuous variables

**Table 2:** Patient and procedural characteristics and association with Gal-3 terciles

Patient characteristics	Overall	1st Tercile	2nd Tercile	3rd Tercile	*p* value
KDIGO					
No AKI	67.9%	73.4%	70.5%	59.6%	< 0.001
Stage 1	26.3%	22.9%	24.6%	31.6%	
Stage 2	4.5%	2.6%	4.2%	6.9%	
Stage 3	1.3%	1.2%	0.8%	1.8%	
Age^a^	65.7 ± 9.9	63.6 ± 10.0	65.5 ± 9.6	66.8 ± 10.6	0.464
Female	22.7%	18.2%	20.6%	32.0%	< 0.001
BMI^a^	29.6 ± 5.5	29.6 ± 5.2	29.6 ± 5.5	29.8 ± 5.9	0.464
BSA^a^	2.0 ± 0.2	2.0 ± 0.3	2.1 ± 0.2	2.0 ± 0.3	0.464
Smoker	21.4%	24.4%	22.2%	21.4%	0.488
Atrial fibrillation	6.5%	5.4%	6.2%	8.7%	0.082
CHF	11.2%	7.3%	8.7%	15.9%	< 0.001
Last pre-op serum creatinine (mean, SD)	1.1 ± 0.6	1.1 ± 0.5	1.1 ± 1.4	1.3 ± 1.0	0.464
Diabetes	38.0%	34.6%	33.9%	43.8%	0.001
Ejection fraction < 40%	12.1%	10.6%	10.0%	12.3%	0.508
Hypertension	81.0%	79.2%	80.1%	83.0%	0.273
IABP pre-op	3.8%	4.6%	4.8%	2.7%	0.165
Prior MI					
No	54.6%	57.3%	57.4%	53.7%	0.098
< 24 h pre-op	1.5%	1.4%	1.5%	2.1%	
> 24 h & < 7 days pre-op	20.5%	18.7%	19.6%	18.0%	
> 7 days & < 365 days pre-op	9.8%	7.9%	8.45%	13.6%	
> 365 days pre-op	13.6%	14.7%	12.7%	12.6%	
Vascular disease	27.8%	26.1%	25.2%	30.2%	0.154
Unstable angina	58.2%	53.7%	55.0%	58.1%	0.347
COPD	12.6%	11.2%	12.1%	15.5%	0.095
Left main, ≥50% stenosis	31.5%	33.0%	35.3%	33.0%	0.669
Prior CABG	2.4%	2.3%	2.8%	1.8%	0.566
Prior PCI	19.6%	20.5%	18.7%	20.0%	0.760
Priority					
Emergent	1.5%	2.5%	1.7%	1.6%	0.815
Urgent	70.1%	68.0%	67.4%	67.8%	
Non-urgent	28.3%	29.5%	30.8%	30.6%	
Received pRBC units		30.4%	35.5%	48.6%	< 0.001
Number of pRBC units given pre-op					
0	97.9%	99.4%	97.5%	97.1%	0.142
1 or more	2.1%	0.6%	2.5%	2.9%	

^a^(Mean, SD)

*AKI* acute kidney injury, *KDIGO* Kidney Disease: Improving Global Outcomes, *BMI*, body mass index (kg/m^2^), *BSA* body surface area (m^2^), *CABG* coronary artery bypass graft, *CHF* congestive heart failure, *COPD* chronic obstructive pulmonary disease, *IABP* intra-operative balloon pump, *MI* myocardial infarction, *PCI* percutaneous coronary intervention, *RBC* red blood cell, *SD* standard deviation

Among patients studied, there was a significant difference between postoperative AKI incidence for older patients and patients with a higher BMI, history of smoking, atrial fibrillation, congrestive heart failure (CHF), diabetes, ejection fraction < 40, prior MI, vascular disease, received transfused blood, and red blood cells transfused postoperatively. There is a significant relationship between elevated Gal-3 measurements and increased AKI severity (*p* < 0.001). For patients in the lowest tercile of Gal-3, 22.9% experienced KDIGO Stage 1 compared to 31.6% in the highest biomarker tercile. Similarly, for patients in the highest Gal-3 tercile, 1.8% of patients experienced KDIGO Stage 3 compared to 1.2% in the lowest tercile.

Table 3 describes the unadjusted and adjusted results for the AKI risk model and Gal-3 biomarker terciles. Before adjustment, patients in the highest tercile of Gal-3 had a 2.86-greater odds of developing postoperative KDIGO Stage 2 or 3 AKI (*p <* 0.001) and 1.70-greater odds of developing KDIGO Stage 1 (*p <* 0.001). After adjustment, patients in the highest tercile had 2.95-greater odds of developing KDIGO Stage 2 or 3 (*p <* 0.001) and 1.71-increased odds of developing KDIGO Stage 1 (*p* = 0.001). When preoperative levels of NT-proBNP were added to the risk prediction model, we observed similar results. Patients in the highest tercile of Gal-3 had 2.85-greater odds of developing KDIGO Stage 2 or 3 AKI (*p =* 0.001) and patients in the lowest tercile at 1.65-greater odds of KDIGO Stage 1 AKI (*p* = 0.003).

**Table 3:** Unadjusted and STS adjusted model evaluating preoperative Gal-3 measurements and association with KDIGO stage severity

	KDIGO Stage 1			KDIGO Stage 2 or 3		
	OR	95% CI	*p* value	OR	95% CI	*p* value
Unadjusted						
Preoperative	1.03	1.01–1.04	0.000	1.04	1.02–1.06	0.000
Natural log	1.47	1.19–1.81	0.000	2.03	1.40–2.94	0.000
Tertiles						
1	1.00	1.00–1.00		1.00	1.00–1.00	
2	1.12	0.83–1.50	0.456	1.36	0.74–2.52	0.322
3	1.70	1.28–2.27	0.000	2.86	1.63–5.01	0.000
Preoperative above median	1.36	1.08–1.72	0.009	2.44	1.53–3.89	0.000
STS Readmission Prediction Model^a^						
Preoperative	1.03	1.01–1.04	0.002	1.03	1.00–1.06	0.005
Natural log	1.40	1.10–1.78	0.006	1.87	1.23–2.86	0.004
Tertiles						
1	1.00	1.00–1.00		1.00	1.00–1.00	
2	1.08	0.79–1.48	0.625	1.37	0.73–2.56	0.329
3	1.71	1.24–2.37	0.001	2.95	1.63–5.34	0.000
Preoperative above median	1.30	1.00–1.68	0.046	2.31	1.39–3.85	0.001
STS Readmission Prediction Model + NT-pro BNP						
Preoperative	1.02	1.01–1.04	0.005	1.03	1.00–1.06	0.007
Natural log	1.31	1.03–1.68	0.028	1.73	1.13–2.65	0.012
Tertiles						
1	1.00	1.00–1.00		1.00	1.00–1.00	
2	1.08	0.79–1.48	0.631	1.36	0.72–2.57	0.337
3	1.65	1.19–2.29	0.003	2.85	1.57–5.16	0.001
Preoperative above median	1.28	0.99–1.66	0.060	2.26	1.35–3.76	0.002

^a^Model adjusts for variables included in the STS readmission prediction model

*KDIGO* Kidney Disease: Improving Global Outcomes, *CI* confidence interval, *OR* odds ratio, *STS* Society of Thoracic Surgeons

The base and augmented models are summarized in Table 4. The base model yieled a c-statistic of 0.69 (95% CI: 0.66–0.71). The base model with the addition of preoperative Gal-3 terciles yielded a c-statistic of 0.70 (95% CI: 0.67–0.72) and has a significant ROCCOMP *p* value of 0.042 compared to the base model alone. With the addition of Gal-3 and NT-proBNP terciles to the base model, the c-statistic remains at 0.70 (95% CI: 0.68 0.73) and is significantly improved from the base model alone (ROCCOMP *p* value = 0.005).

**Table 4:** Model comparison statistics evaluating the discriminatory power of the base regression model and the additive value of preoperative Gal-3 terciles and preoperative NT-proBNP terciles

	C-statistic (95% CI)	ROCCOMP *p* value^a^
STS Readmission Prediction Model	0.69 (0.66–0.71)	
STS model + Gal-3 preoperative terciles	0.70 (0.67–0.72)	0.042
STS model + combined Gal-3 and NT-pro BNP preoperative terciles	0.70 (0.68–0.73)	0.005

^a^ROC comparison against base model

In an exploratory analysis where we compared preoperative Gal-3 terciles to preoperative eGFR values, we did not find an appreciable difference between the two makers and risk of developing AKI. Models comparing preoperative Gal-3 tercile values to continuous eGFR values are reported in Table 5.

**Table 5:** Model evaluation Gal3 & eGFR

	STS Readmission Prediction Model + Risk Marker					
	C-statistic (95% CI)	NRI	NRI *P*	IDI	IDI *P*	Test of Equality *P*^b^
STS Readmission Model	0.69 (0.66–0.71)					
Preoperative Gal-3 terciles	0.70 (0.67–0.72)	0.03	0.067	0.01	0.000	0.042
Preoperative eGFR^a^ (mL/min/1.73 m^2)	0.69 (0.66–0.72)	0.02	0.124	0.00	0.010	0.302

*P* represents the statistical *p* value.

^a^Estimated glomerular filtration rate (eGFR)

^b^ROC comparison against base model

*NRI* Net Reclassification Improvement index, *IDI* Integrated Discrimination Improvement index

## Discussion

We are the first to demonstrate a significant relationship between the inflammatory biomarker Gal-3 and AKI in a multi-site, prospectively enrolled cohort of patients undergoing cardiac surgery. We found Gal-3 concentrations increased concurrently with decreasing kidney function. In our study, patients in the highest tercile of preoperative Gal-3 levels had 1.7 times the adjusted odds of KDIGO Stage 1 AKI compared to patients in the lowest tercile of Gal-3. Patients in the highest tercile of preoperative Gal-3 also had 2.9 times the adjusted odds of KDIGO Stage 2 or 3 AKI compared to the lowest tercile.

Gal-3 is a well-established biomarker for cardiac fibrosis, ventricular dysfunction, and poor prognosis in heart failure [21–23]. In addition, Gal-3 has demonstrated diagnostic and prognostic value in diseases of the kidney [9, 11, 18]. Drechsler et al. found a positive association between elevated levels of Gal-3 and adverse outcomes in patients with preexisting renal disease. Additionally, in the well recognized Framingham Heart Study, researchers demonstrated that elevated Gal-3 levels precede the development of CKD [10, 11]. Prior to our analysis, studies examining the relationship between Gal-3 and acute renal injury after surgery had been limited to animal models. Multiple animal studies have demonstrated that Gal-3 expression is up-regulated in the kidneys in response to ischemic and toxic injury and is associated with renal fibrosis [14–17].

In the kidney, Gal-3 has multiple functions including regulating the inflammatory response and cell growth, proliferation, and differentiation [24, 25]. Gal-3 has been proposed to be a marker of combined cardiac and renal fibrosis in the chronic setting [24, 25]. The prognostic value of baseline impaired cardiac and renal functional reserve may predict risk of AKI after cardiac surgery [26]. A 15% change from baseline has been associated with significantly more heart failure hospitalizations and increased mortality compared with lower and decreasing levels [21]. In addition, Gal-3 has been shown to stimulate macrophages to release pro-inflammatory cytokines (e.g. MCP-1, IL-6, and IL-1B) and produce reactive oxygen species, enhancing the inflammatory response in the kidney [17]. Knocking-out the Gal-3 gene or directly inhibiting the Gal-3 protein is known to inhibit renal fibrosis and lessen renal injury in AKI [16, 27, 28].

We have demonstrated that preoperative levels of circulating Gal-3 are associated with AKI and AKI severity after cardiac surgery. Patients with higher levels of circulating Gal-3 may be predisposed to excessive inflammatory processes. Preoperative Gal-3 levels could also be acting as a marker for early CKD, identifying patients more susceptible to AKI because of underlying kidney disease. Further, preoperative Gal-3 may be serving as an indicator of heart failure (HF) and those patients at risk of AKI due to ischemic renal injury secondary to pump failure. Preoperative measurement of Gal-3 may provide a means to evaluate AKI risk due to multiple etiologies.

Multiple preoperative biomarkers have been evaluated for their ability to predict AKI after cardiac surgery. Cystatin C (CysC) is a circulating protease inhibitor and correlates with the glomerular filtration rate (GFR) [13]. Its preoperative values have been shown to independently associate with AKI following cardiac surgery [29, 30]. Similarly, prior research has demonstrated a relationship between brain natriuretic peptide (BNP), NT-proBNP and Gal-3 with elevation in both markers related to outcomes [31]. Preoperative BNP, a polypeptide released by the ventricles in response to volume overload, has been shown to predict postoperative development of AKI [29, 32]. The inclusion of NT-proBNP in our study resulted in only a moderate difference from our adjusted prediction model. Compared to the adjusted model, the above median preoperative Gal3 measurements were non-signfiicant for those developing KDIGO Stage 1 in augmented model with NT-proBNP. The inclusion of BNP in our prediction model could provide important inferences on cardiac-surgery associated with AKI and heart failure, but further evaluation is needed. Given the varying kinetics and characteristics of individual biomarkers, it is likely that the measurement of multiple biomarkers, in addition to Gal-3, is necessary to accurately perform preoperative risk assessment for AKI [29, 33].

The primary strength of this study is its large sample size comprised of patients who underwent cardiac surgery at eight hospitals across Maine, Vermont and New Hampshire. Studies on preoperative biomarker levels and prediction of AKI have been previously conducted with small cohorts, in single-center settings and lacked defined, explicit outcomes [34]. In this study, we leveraged the NNECDSG registry, which is comprehensive in the patient and procedural data that it contains. The completeness and quality of this data also helps ensure that adequate adjustment was carried out.

### Study limitations

There are limitations to this study to consider. First, we lacked detailed information on some conditions known to affect the incidence of AKI including cardiopulmonary bypass times, hemodynamics or the use of inotropic and vasoactive drugs in the perioperative period. Therefore, residual confounding of the demonstrated association of Gal-3 with AKI can not be excluded. Gal-3 has been found to be correlated with pre-existing renal disease and heart failure. Medical support to maintain blood pressure arguably would be more aggressive in patients with higher Gal-3 values, and our results would more likely be biased towards the null-hypothesis. Secondly, we also used creatinine-based definitions for AKI which are relatively insensitive and non-specific in the period directly following insults to the kidney [35]. Thirdly, we were also unable to evaluate long-term outcomes such as major adverse renal and cardiac events (MARCE). Fourthly, given the unique patient characteristics associated with the CABG patient population, there may be reproducibility limitations with a more heterogeneous population. Finally, the mean imputation method used to address missing data may influence the overall composition and performance of the prediction model.

## Conclusion

Improving the predictive ability of AKI risk prior to surgery is critical to take preventative measures and counsel patients on potential outcomes after cardiac surgery. Elevated preoperative Gal-3 levels may be used to augment risk information for patients at greatest risk of developing AKI and AKI severity after cardiac surgery. If Gal-3 is elevated, there are several AKI mitigation strategies to employ including avoiding surgery on the same day as cardiac catherization, limiting transfusion, remote ischemic preconditioning prior to surgery and stopping angiotensin-converting enzymes inhibitors (ACEIs) and angiotensin receptor blockers (ARBs) for 2 days after surgery.

We are the first to demonstrate a significant association between the inflammatory and fibrosis biomarker Gal-3 and AKI in patients undergoing cardiac surgery. Our findings suggest that preoperative Gal-3 levels could be used to identify patients at the highest risk of developing AKI after cardiac surgery.

## Appendix

**Table 6:** STS Model Variables and NNE Registry Data

STS	NNE
1. We were unable to adjust for chronic lung disease or prior myocardial infarction in the same way as the investigators did in the STS preoperative readmission model.	
STS registry had data on the severity of chronic lung disease (none, mild, moderate, severe)	NNE registry only contains information on whether or not members of our patient cohort had chronic obstructive pulmonary disease (COPD) or not.
2. The STS and NNE registries also categorize prior myocardial infarctions in different ways.	
STS uses four different categories (no recent MI, MI between one and 21 days ago, MI more than six and less than 24 h ago, and MI less than or equal to 6 h ago)	NNE registry instead uses five categories for our cohort (no prior MI, MI less than 24 h prior to operation, MI more than 24 h but less than 7 days prior to operation, MI more than 7 days but less than 1 year prior to operation, and MI more than 0 year prior to operation)
3. We were unable to adjust for immunosuppressive treatment at all, since the NNE registry did not collect that information for our cohort.	
4. Our final NNE version of the STS preoperative readmission risk adjustment model included 30 covariates.	

This table describes the differences in variables between the STS model and variables available in the NNE registry dataset

## Abbreviations

ACEIs: Angiotensin-converting enzymes inhibitors
AKI: Acute kidney injury
AKIN: Acute Kidney Injury Network
AMI: Acute myocardial infarction
ARB: Angiotensin receptor blockers
BMI: Body mass index
BSA: Body surface area
CABG: Coronary artery bypass graft
CHF: Chronic heart failure
CI: Confidence interval
COPD: Chronic obstructive pulmonary disease
IABP: Intra-aortic balloon pump
IL-1: Interleukin-1
KDIGO: Kidney Disease: Improving Global Outcomes
MARCE: Major adverse renal and cardiac events
MI: Myocardial infarction
NNE: Northern New England
NNECDSG: Northern New England Cardiovascular Disease Study Group
OR: Odds ratio
PCI: Percutaneous coronary intervention
pRBC: Packed red blood cells
ST2: Serum soluble ST2
STS: Society of Thoracic Surgeons
VAD: Ventricular assist device
